# Impact of waning immunity against SARS-CoV-2 severity exacerbated by vaccine hesitancy

**DOI:** 10.1371/journal.pcbi.1012211

**Published:** 2024-08-05

**Authors:** Chadi M. Saad-Roy, Sinead E. Morris, Mike Boots, Rachel E. Baker, Bryan L. Lewis, Jeremy Farrar, Madhav V. Marathe, Andrea L. Graham, Simon A. Levin, Caroline E. Wagner, C. Jessica E. Metcalf, Bryan T. Grenfell

**Affiliations:** 1 Miller Institute for Basic Research in Science, University of California, Berkeley, California, United States of America; 2 Department of Integrative Biology, University of California, Berkeley, California, United States of America; 3 Department of Pathology and Cell Biology, Columbia University Medical Center, Columbia University, New York, New York, United States of America; 4 Department of Biosciences, University of Exeter, Penryn, United Kingdom; 5 Department of Epidemiology, Brown School of Public Health, Brown University, Providence, Rhode Island, United States of America; 6 Network Systems Science and Advanced Computing Division, Biocomplexity Institute, University of Virginia, Charlottesville, Virginia, United States of America; 7 The Wellcome Trust, London, United Kingdom; 8 Department of Computer Science, University of Virginia, Charlottesville, Virginia, United States of America; 9 Department of Ecology and Evolutionary Biology, Princeton University, Princeton, New Jersey, United States of America; 10 Department of Bioengineering, McGill University, Montreal, Canada; 11 School of Public and International Affairs, Princeton University, Princeton, New Jersey, United States of America; US Army Medical Research and Materiel Command: US Army Medical Research and Development Command, UNITED STATES OF AMERICA

## Abstract

The SARS-CoV-2 pandemic has generated a considerable number of infections and associated morbidity and mortality across the world. Recovery from these infections, combined with the onset of large-scale vaccination, have led to rapidly-changing population-level immunological landscapes. In turn, these complexities have highlighted a number of important unknowns related to the breadth and strength of immunity following recovery or vaccination. Using simple mathematical models, we investigate the medium-term impacts of waning immunity against severe disease on immuno-epidemiological dynamics. We find that uncertainties in the duration of severity-blocking immunity (imparted by either infection or vaccination) can lead to a large range of medium-term population-level outcomes (*i.e*. infection characteristics and immune landscapes). Furthermore, we show that epidemiological dynamics are sensitive to the strength and duration of underlying host immune responses; this implies that determining infection levels from hospitalizations requires accurate estimates of these immune parameters. More durable vaccines both reduce these uncertainties and alleviate the burden of SARS-CoV-2 in pessimistic outcomes. However, heterogeneity in vaccine uptake drastically changes immune landscapes toward larger fractions of individuals with waned severity-blocking immunity. In particular, if hesitancy is substantial, more robust vaccines have almost no effects on population-level immuno-epidemiology, even if vaccination rates are compensatorily high among vaccine-adopters. This pessimistic scenario for vaccination heterogeneity arises because those few individuals that are vaccine-adopters are so readily re-vaccinated that the duration of vaccinal immunity has no appreciable consequences on their immune status. Furthermore, we find that this effect is heightened if vaccine-hesitants have increased transmissibility (*e.g*. due to riskier behavior). Overall, our results illustrate the necessity to characterize both transmission-blocking and severity-blocking immune time scales. Our findings also underline the importance of developing robust next-generation vaccines with equitable mass vaccine deployment.

## Author summary

While the SARS-CoV-2 outbreak continues, the deployment of vaccines in many regions has blunted the severity of SARS-CoV-2 infections and decreased hospitalizations. However, the medium-term impacts of the duration of severity-blocking immunity, and its potential interactions with heterogeneous vaccine uptake (*e.g*. from vaccine hesitancy) or more robust vaccines, remain unknown. To titrate these effects, we use immuno-epidemiological models to examine potential future scenarios. We find that sufficient vaccine hesitancy (and correspondingly higher vaccination rates among adopters) can rapidly increase the fraction of individuals infected after waned severity-blocking immunity even when robust vaccines are deployed. This result underlines that pharmaceutical developments for broadly protective vaccines should be combined with campaigns to increase vaccine uptake globally. We also show that this fraction is highly dependent on underlying immune uncertainties, which illustrates the importance of accurately measuring immune parameters for proper prediction based on hospitalization data.

## Introduction

The severe acute respiratory syndrome coronavirus 2 (SARS-CoV-2) pandemic is a public health emergency that has had a dramatic impact across the world. In turn, it has generated a mass of epidemiological data and led to large modelling efforts [1]. Initially, guided by data analyses, a number of jurisdictions successfully implemented a range of control measures to decrease transmission, prevent a surge in infections, and decrease the burden on healthcare systems (*e.g*. see [2] for a retrospective analysis). In parallel, research into pharmaceutical measures (such as vaccination or therapeutics) began, with hopes to eventually control SARS-CoV-2 transmission via vaccination. Notably, with high enough coverage, transmission-blocking vaccines (*i.e*. that elicit immunity against infection) could lead to effective control and local elimination [3–6]. However, while the development of safe vaccines was successful (*e.g*. [7–9]), the susceptibility of vaccinated individuals to breakthrough infection relatively soon after vaccination (*e.g*. even within weeks [10]) in conjunction with the emergence of immune-escape variants (*e.g*. [11]) indicates that local elimination is not possible with the current generation and partial uptake of vaccines. Since the deployment of these vaccines, many jurisdictions have changed their approach for SARS-CoV-2 management to focus on mitigation against severe infections via vaccination.

Since the onset of the pandemic, a number of important gaps in our understanding of SARS-CoV-2 epidemiology have been addressed by models [1]. For example, future transmission dynamics were illuminated in an early landmark paper by Kissler et al. [12]; the role of climate and susceptibility on pandemic dynamics was investigated by Baker et al. [13, 14]; Lavine et al. [15] clarified the path to endemicity and the role of age structure; and others examined the role of novel variants [16–18]. In our previous work, we have investigated many SARS-CoV-2 immuno-epidemiological uncertainties from a qualitative perspective. First, we used and extended a simple SIR(S) model (see [19]) to show that the relative susceptibility to infection after waning of total transmission-blocking immunity *ε* (so that *ε* = 0 and *ε* = 1 reduce to the SIR and SIRS models, respectively, and thus *ε* is a proxy for the “strength of immunity”) is a key determinant of post-pandemic trajectories [6]. We then extended this framework to incorporate two-dose vaccines [20], investigate the potential effects of vaccine nationalism [21], and examine the impact of accumulating immunity on the potential future burden of chronic disease [22].

However, a number of key immuno-epidemiological questions remain. At the heart of these are uncertainties in waning immunity against severe disease (*i.e*. ‘severity-blocking immunity’), and the ensuing potential outcomes in the medium term. In particular, from a public health standpoint, determining the likelihood, timing, and magnitude of the next surge in severe disease is crucial. Furthermore, many regions now rely on hospitalizations to monitor infection levels (especially with the pause of the ONS COVID-19 infection survey study in the UK); waning severity-blocking immunity could have an important effect on these dynamics, *e.g*. variations in the fraction of infections that require hospitalization at a given time, and thus crucially affect subsequent inferences. Additionally, since we have determined that the strength of immunity is a central parameter that shapes medium-term immuno-epidemiological dynamics [6], another outstanding unknown is the potential interplay between this parameter and the duration of severity-blocking immunity. Finally, given important developments toward mucosal vaccines [23–25], a major question is to determine the impacts that such vaccines with long-lasting transmission-blocking protection could have on potential outcomes and their uncertainties. For example, reducing these uncertainties may be important for robust estimates of infection levels and epidemic dynamics.

**Fig 1:**
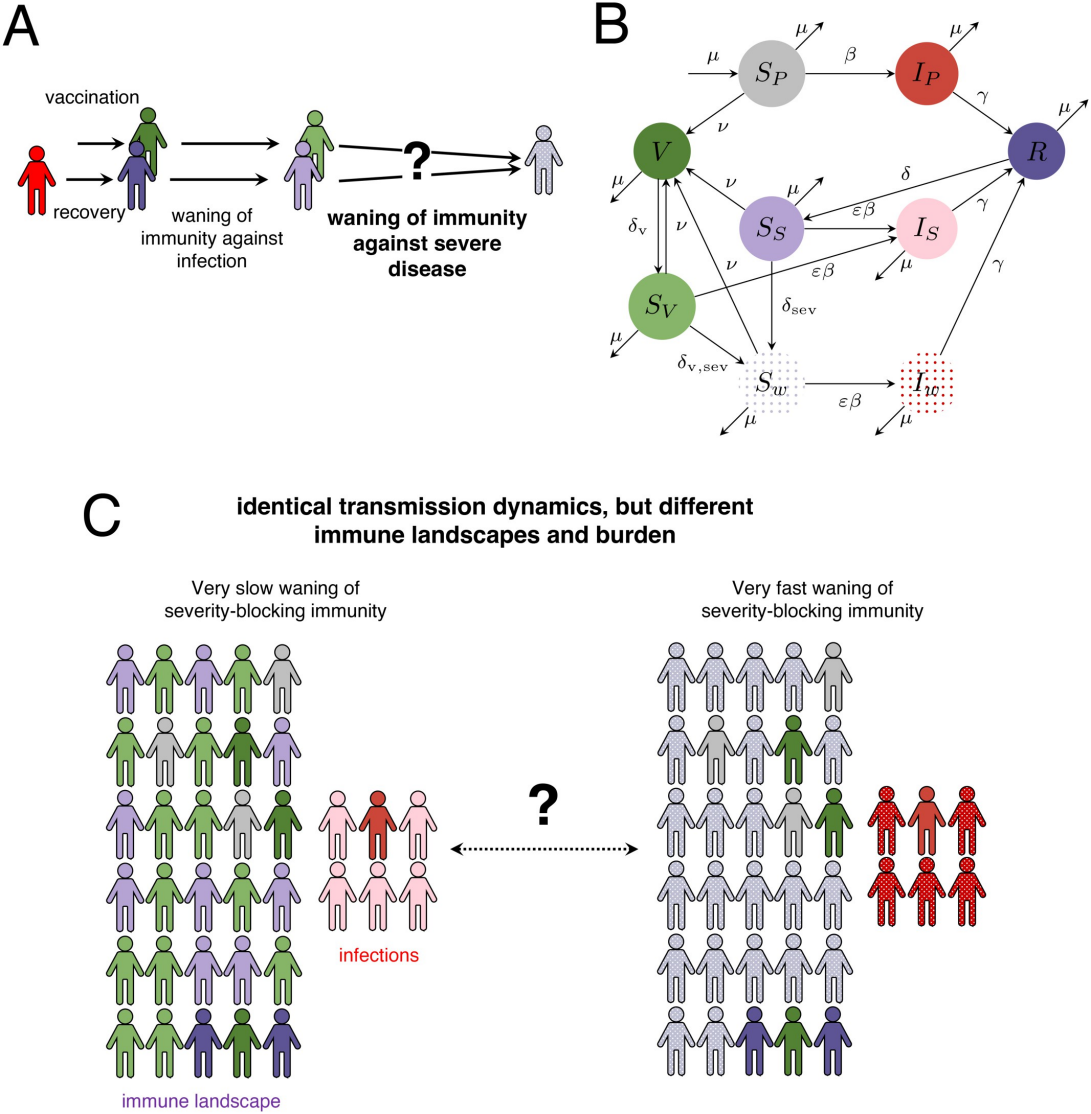
Model formulation. (*A*) Schematic of individual immunity progression after infection or vaccination. (*B*) Model flow diagram, extended from Fig 3A of [6]. Each colour denotes an infection or immunity class. (*C*) Schematic of the range of population-level outcomes based on severity-blocking immunity.

In this paper, we extend previous modelling efforts [6] to include a timescale of waning immunity against severe disease (Fig 1A). We begin with a characterization of the interplay between the strength of immunity, average duration of severity-blocking immunity, and vaccination rate, and their respective (and combined) impacts on infection levels in individuals with waned severity-blocking immunity. We then investigate the impact of vaccine characteristics on these dynamics, and we examine potential synoptic immuno-epidemiological landscapes. Finally, we extend our model to include heterogeneities in vaccination that are driven by unequal access or hesitancy. While we cast our results in terms of vaccine hesitancy for simplicity, our findings are broadly applicable for any setting with heterogeneous uptake in vaccination.

## Model framework

We extend the model of [6]. As in [6], *S*_*P*_ denotes the fraction of fully susceptible individuals, *I*_*P*_ and *I*_*S*_ denotes the fraction of individuals with primary and secondary infections (the latter which have relative transmissibility *α*), respectively, *R* denotes the fraction of individuals that have recovered and are fully immune, *S*_*S*_ denotes the fraction of individuals with waned transmission-blocking immunity and who have relative susceptibility *ε* to infection, and *V* denotes the fraction of individuals that have been vaccinated and are fully immune. Furthermore, again as in [6] *μ* is the birth/death rate, *γ* is the recovery rate, *δ* and *δ*_*V*_ are the rates of waning of natural and vaccinal transmission-blocking immunity, respectively, and *β* is the transmission rate (assumed to be time-dependent [13] as in previous work [6]). We additionally denote *S*_*w*_ as the fraction of individuals with waned severity-blocking immunity, and *I*_*w*_ as the fraction of individuals with infection after waned severity-blocking immunity. We denote *δ*_sev_ and *δ*_*V*,sev_ as the rates of waning from *S*_*S*_ to *S*_*w*_ and from *S*_*V*_ to *S*_*w*_, respectively. The equations are as follows:

$$\begin{array}{c} \frac{dS_{P}}{dt}=\mu -\beta \left(t\right)S_{P}\left(I_{P}+\alpha I_{S}+\alpha I_{w}\right)-\mu S_{P}-s_{\text{vax}}\nu S_{P}, \end{array}$$ (1a)

$$\begin{array}{c} \frac{dI_{P}}{dt}=\beta \left(t\right)S_{P}\left(I_{P}+\alpha I_{S}+\alpha I_{w}\right)-\left(\gamma +\mu \right)I_{P}, \end{array}$$ (1b)

$$\begin{array}{c} \frac{dR}{dt}=\gamma \left(I_{P}+I_{S}+I_{w}\right)-\left(\delta +\mu \right)R, \end{array}$$ (1c)

$$\begin{array}{c} \frac{dS_{S}}{dt}=\delta R-\varepsilon \beta \left(t\right)S_{S}\left(I_{P}+\alpha I_{S}+\alpha I_{w}\right)-\mu S_{S}-s_{\text{vax}}\nu S_{S}-\delta_{\text{sev}}S_{S}, \end{array}$$ (1d)

$$\begin{array}{c} \frac{dI_{S}}{dt}=\varepsilon \beta \left(t\right)S_{S}\left(I_{P}+\alpha I_{S}+\alpha I_{w}\right)+\varepsilon \beta \left(t\right)S_{V}\left(I_{P}+\alpha I_{S}+\alpha I_{w}\right)-\left(\gamma +\mu \right)I_{S}, \end{array}$$ (1e)

$$\begin{array}{c} \frac{dV}{dt}=s_{\text{vax}}\nu \left(S_{P}+S_{S}+S_{w}+S_{V}\right)-\left(\mu +\delta_{V}\right)V, \end{array}$$ (1f)

$$\begin{array}{c} \frac{dS_{V}}{dt}=\delta_{V}V-\varepsilon \beta \left(t\right)S_{V}\left(I_{P}+\alpha I_{S}+\alpha I_{w}\right)-\left(\delta_{V,\text{sev}}+\mu \right)S_{V}-s_{\text{vax}}\nu S_{V}, \end{array}$$ (1g)

$$\begin{array}{c} \frac{dS_{w}}{dt}=\delta_{V,\text{sev}}S_{V}+\delta_{\text{sev}}S_{S}-\varepsilon \beta \left(t\right)S_{w}\left(I_{P}+\alpha I_{S}+\alpha I_{w}\right)-\mu S_{w}-s_{\text{vax}}\nu S_{w}, \end{array}$$ (1h)

$$\begin{array}{c} \frac{dI_{w}}{dt}=\varepsilon \beta \left(t\right)S_{w}\left(I_{P}+\alpha I_{S}+\alpha I_{w}\right)-\left(\mu +\gamma \right)I_{w}. \end{array}$$ (1i)

Note that we set *α* = 1 throughout and focus on *ε* (see [6, 20–22]).

Thus, after recovery or vaccination, individuals have a period of “complete” immunity (*R* or *V*, respectively), after which they wane into partially susceptible classes (*S*_*S*_ and *S*_*V*_, respectively), where their relative susceptibility to infection is *ε*. Beyond these classes, individuals eventually wane to *S*_*w*_ (at rates *δ*_sev_ and *δ*_*V*,sev_, respectively), which denotes individuals with waned severity-blocking immunity. In this class, the relative susceptibility to infection is still *ε*, but individuals enter a different infectious class, *I*_*w*_, if they are infected after such waning (see Fig 1B for flow diagram). Thus, individuals have severity-blocking immunity while they are in *R* and *S*_*S*_, or in *V* and *S*_*V*_ (with average durations $\frac{1}{\delta }+\frac{1}{\delta_{\text{sev}}}$ and $\frac{1}{\delta_{V}}+\frac{1}{\delta_{V,\text{sev}}}$, respectively).

To focus on clinical severity-blocking immunity, note that we assume that the relative susceptibility of individuals in *I*_*w*_ and *I*_*S*_ are the same (and that the relative transmissibility *α* = 1 in *I*_*w*_ and *I*_*S*_ is also identical). Because of this assumption, our model interpolates between a range of immune landscapes and population-level burden, while having identical transmission dynamics across scenarios (schematically depicted in Fig 1C). In one extreme case, if there is no (or very slow) waning of severity-blocking immunity, no individuals enter the compartments with waned severity-blocking immunity. On the other hand, if there is very fast waning of severity-blocking immunity, all individuals enter *S*_*w*_ almost immediately after complete transmission-blocking immunity wanes. Additionally, since hospitalizations (in the longer term) are likely to reflect the dynamics of *I*_*w*_, we also calculate the fraction of individuals that are in this class over time (*i.e*. $\frac{I_{w}}{I_{P}+I_{S}+I_{w}}$). Note that the incorporation of vaccine hesitancy in our model is described in the S1 Text, *electronic supplementary materials*. Finally, we have produced an online interactive application (at https://grenfelllab.shinyapps.io/covid19immunity/), which can be used to examine a broad set of model scenarios.

## Results and discussion

### Vaccination, duration of severity-blocking immunity, and dynamics of waned infections

**Fig 2:**
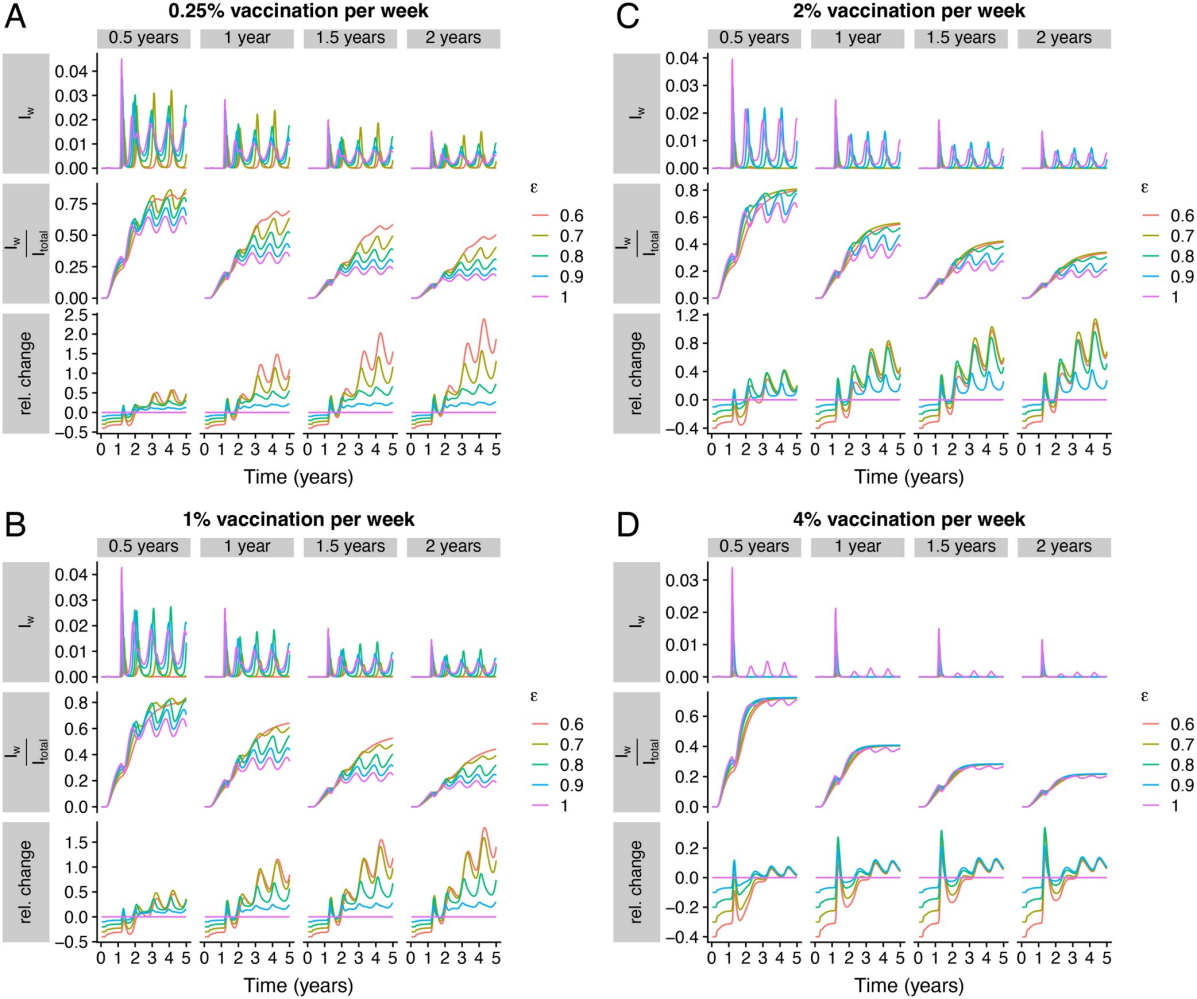
Dynamics of different durations of natural and vaccinal severity protection, with variable vaccination rates, for different strengths of immunity. (*A*), (*B*), (*C*), and (*D*) have vaccination rates *ν* = 0.0025 per week, *ν* = 0.01 per week, *ν* = 0.02 per week, and *ν* = 0.04 per week, respectively. In all panels, we assume that $\frac{1}{\delta }=0.25$ years and that $\frac{1}{\delta_{V}}=0.33$ years. For each column, we assume that the duration of severity-blocking immunity imparted from vaccination or infection is the same and is equal to the columnar label *ℓ*_*c*_, *i.e*. $\frac{1}{\delta }+\frac{1}{\delta_{\text{sev}}}=\ell_{c}$ and $\frac{1}{\delta_{V}}+\frac{1}{\delta_{V,\text{sev}}}=\ell_{c}$. Thus, $\frac{1}{\delta_{\text{sev}}}=\left(\ell_{c}-0.25\right)$ years and $\frac{1}{\delta_{V,\text{sev}}}=\left(\ell_{c}-0.33\right)$ years. In each panel, the *top*, *middle*, and *bottom* rows depict the fraction of individuals in *I*_*w*_, the fraction of infections that are in *I*_*w*_ (*i.e*. $\frac{I_{w}}{I_{\text{total}}}$, where $I_{\text{total}}=I_{P}+I_{S}+I_{w}$), and the relative change in $f_{\varepsilon }\left(t\right)=\frac{I_{w}\left(t\right)}{I_{\text{total}}\left(t\right)}$ for each *ε* compared to *ε* = 1, *i.e*. $\frac{f_{\varepsilon }\left(t\right)-f_{1}\left(t\right)}{f_{1}\left(t\right)}$, respectively (for weeks when *f*_1_(*t*) > 0). Other parameters are $\gamma =\frac{7}{5}$ week^−1^ and *μ* = 0.02 years^−1^, as in previous work [6, 20–22]. The initial conditions here and throughout are a fraction 10^−9^ of individuals with primary infection (*I*_*P*_) and the remainder fully susceptible (*S*_*P*_), which is as in previous work with the simpler model [6].

In Fig 2, we examine the potential epidemiological dynamics that result in changes of both severity-blocking immunity duration and vaccination coverage. We use seasonal transmission rates as in previous work [6, 22], and assume the same simple nonpharmaceutical intervention settings as in [22]. (For a specific expression for *β*(*t*) in the absence of nonpharmaceutical intervention, see [6], which uses values derived by [13].) For each vaccination rate (*i.e*. each panel of Fig 2), we plot the fraction of individuals that are infected with waned severity-blocking immunity (*i.e*. *I*_*w*_) (*top row*), the fraction of all infections that *I*_*w*_ represents (*i.e*. $\frac{I_{w}}{I_{\text{total}}}$) (*middle row*), and the relative change in this latter fraction compared to complete susceptibility to reinfection after transmission-blocking immunity wanes (*i.e*. *ε* = 1 giving the SIRS model, see the caption of Fig 2 for mathematical details) (*bottom row*). For all panels of Fig 2, we assume the same average duration of transmission-blocking vaccinal (0.33 years) and natural (0.25 years) immunity. Note that while we take these to be relatively short (as in [22]) since infection can happen relatively rapidly after recovery or vaccination (*e.g*. for infection after vaccination see [10]), the effects of longer durations can be examined thoroughly with our companion interactive online application (at https://grenfelllab.shinyapps.io/covid19immunity/). Furthermore, in each panel of Fig 2, we assume that the durations of vaccinal and natural severity-blocking immunity are the same across a column (identified by the columnar label). Additionally, note that since the vaccination rate is fixed within each panel, the transmission dynamics across columns of each panel for a fixed value of *ε* are identical. However, because of the additional immune time scale, the underlying immunity landscapes change, leading to differences in infection characteristics and potential burden.

Intuitively, as the duration of severity-blocking immunity increases, the fraction of individuals with infections after severity-blocking immunity has waned decreases (compare left to right plots of the top rows of Fig 2A–2D). Similarly, driven by more frequent boosting of immunity, higher vaccination rates result in further decreases (compare top rows of Fig 2A–2D). Furthermore, a lower relative susceptibility to reinfection (*i.e*. stronger immunity) initially leads to a smaller fraction of infections with waned severity (*middle rows*, Fig 2A–2D). However, especially for lower vaccination rates, an increase in the strength of immunity (lower *ε*) can potentially lead to larger fractions of infections that are in *I*_*w*_ (*middle* and *bottom rows*, Fig 2A). Additionally, intermediate values of *ε* can lead to larger peaks (and deeper troughs) in *I*_*w*_ (*top row*, Fig 2A). Interestingly, these results are partially reminiscent of the findings of [6], where, in some scenarios, stronger immunity can lead to a bigger (and delayed) second peak in infections. Finally, very high vaccination coverage (Fig 2) dampens these effects because of more frequent gains in immunity.

Overall, these results illustrate an additional potential complication associated with predicting the number of total infections based on hospitalizations alone, in addition to a wide variety of known difficulties. To reduce this particular complexity, such predictions would likely necessitate robust parameter estimates for the strength of immunity and the duration of severity-blocking immunity. These could be obtained from large immuno-epidemiological cohort studies, echoing previous calls for such monitoring [26–29].

### Future vaccine refinements

**Fig 3:**
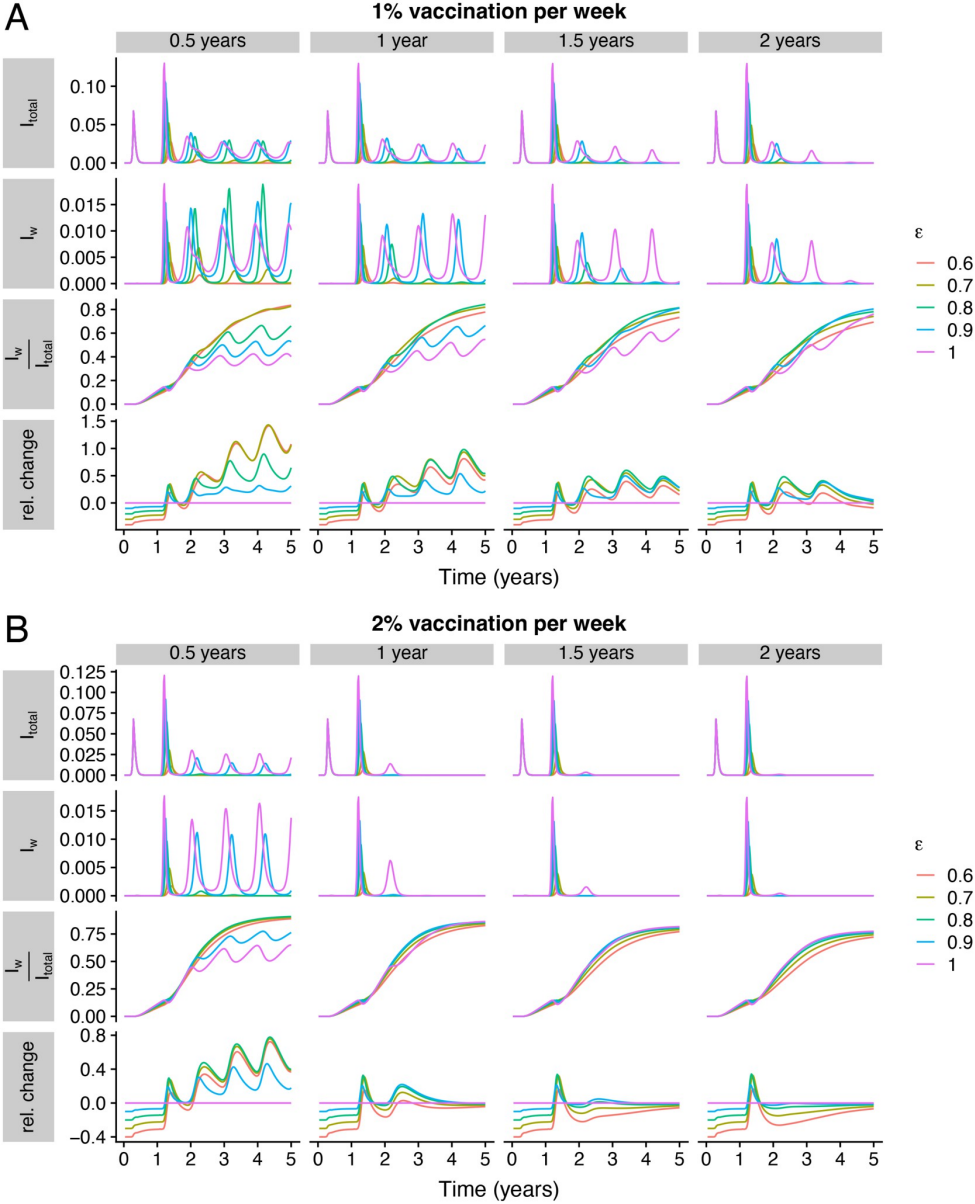
Impacts of longer transmission-blocking vaccines on severity dynamics. In (*A*) and (*B*), the vaccination rates are 0.01 per week and 0.02 per week, respectively. In both panels, the top row denotes the total fraction *I*_total_ = *I*_*P*_ + *I*_*S*_ + *I*_*w*_ of individuals that are infected. The second to fourth rows are as in the rows of each panel of Fig 2 (see caption of Fig 2 for definitions). Across both panels, we assume that the duration of vaccinal transmission-blocking immunity is 90% of the duration of severity-blocking immunity (the columnar label), and that transmission-blocking and severity-blocking immunity after infection last 0.25 years and 1.5 years, respectively (*i.e*. $\frac{1}{\delta }=0.25$ years and $\frac{1}{\delta }+\frac{1}{\delta_{\text{sev}}}=1.5$ years).

So far, we have assumed that the period of complete immunity imparted by vaccination is transient, with relatively high susceptibility after waning. While this reflects current settings with existing vaccines (*e.g*. in part due to circulating immune-escape variants), pan-coronavirus and pan-sarbecovirus vaccines [30] are in development. Additionally, there have been recent landmark advances in the development of mucosal vaccines [23, 24], which would likely be able to more successfully block transmission. Furthermore, it seems that such a mucosal vaccine could generate immunity across sarbecoviruses [23], and thus potentially generate broad immune responses to novel SARS-CoV-2 variants. In Fig 3, we examine the impact of a more durable transmission-blocking vaccine on severity dynamics, for intermediate (1% per week) and high (2% per week) vaccination rates (panels *A* and *B*, respectively). To allow for appropriate comparisons within and across panels, we assume that the duration of vaccinal severity-blocking immunity is conserved within a column (indicated by the columnar label) within each panel. Furthermore, we assume that for 90% of that duration, vaccinal immunity also fully blocks transmission. (Note that this contrasts with Fig 2, where the average complete vaccinal immunity was assumed to be 0.33 years.) Finally, we take a moderately optimistic assumption and assume that severity-blocking immunity after infection lasts on average 1.5 years (in Fig 2, this value was varied concurrently with that imparted following vaccination). Finally, in each panel, the top row denotes the total infections over time, and the bottom three rows are as in those of each panel of Fig 2.

Even for an intermediate vaccination rate, a more durable vaccine leads to fewer total infections (Fig 3, *top row*, compare left to right plots) and fewer infections after severity-blocking immunity has waned (*second row*). Furthermore, with increases in durability, the fraction $\frac{I_{w}}{I_{\text{total}}}$ depends increasingly less on the strength of immunity (*third and bottom rows*, Fig 3, compare left to right plots). In a different setting, this decrease in dependence on *ε* is akin to that observed in Fig 2 for very high vaccination rates. Intuitively, a more durable vaccine is analogous to very high vaccination rates (*i.e*. more frequent boosting) with a less durable vaccine. Finally, a high vaccination rate further accentuates the effects of a durable vaccine on epidemiological dynamics (compare Fig 3A and 3B). Thus, the development and deployment of a durable vaccine, combined with a high vaccination rate, can substantially reduce uncertainties in outcomes.

### Immuno-epidemiological outlooks

**Fig 4:**
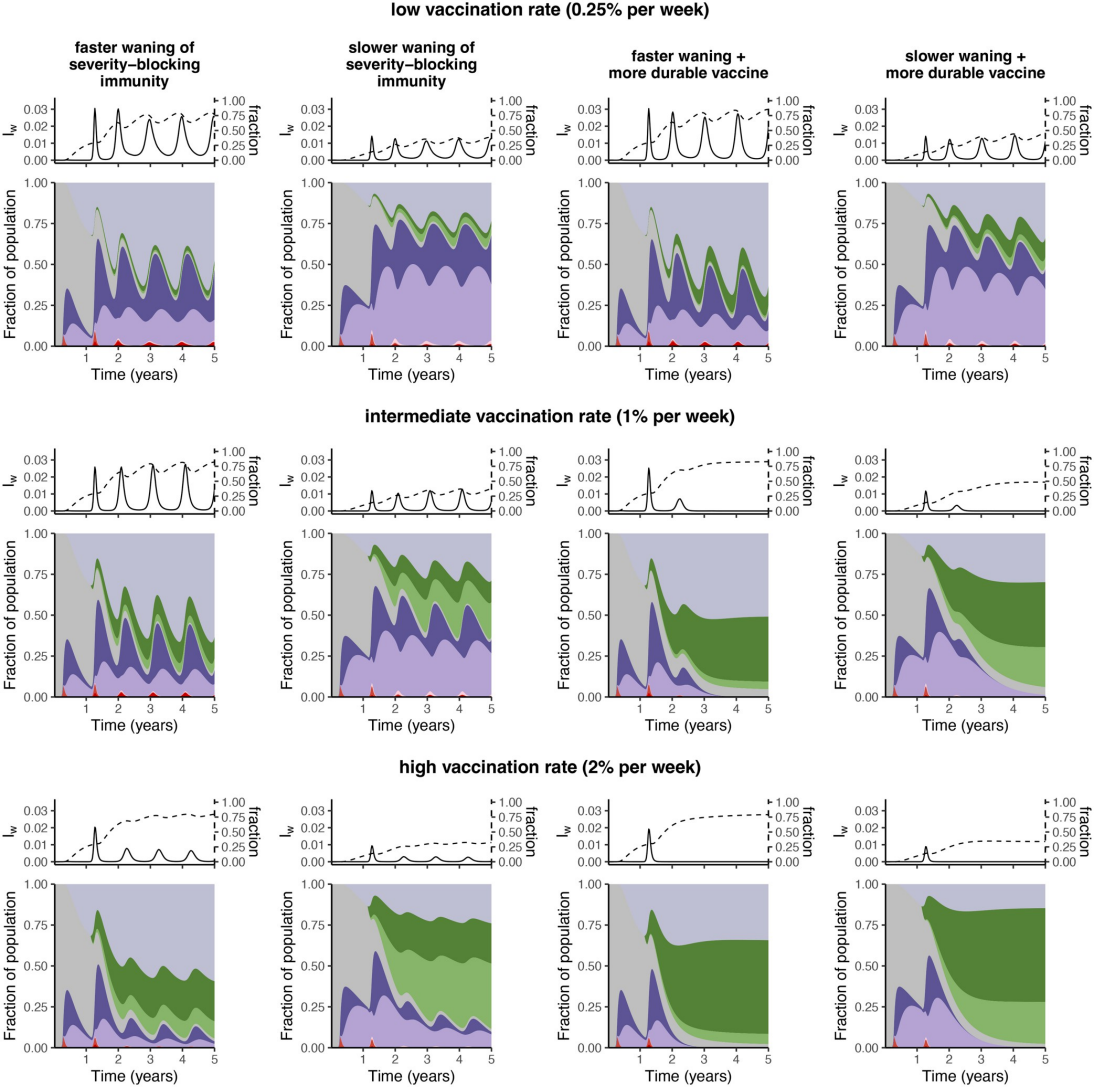
Synoptic landscapes of severity-blocking immunity. The *top*, *middle* and *bottom* rows have vaccination rates 0.0025, 0.01, and 0.02 per week, respectively. The *leftmost* two columns illustrate scenarios with a less durable vaccine, *i.e*. $\frac{1}{\delta_{V}}=0.33$ years, whereas the *rightmost* two columns represent scenarios with a more durable vaccine, *i.e*. $\frac{1}{\delta_{V}}=1.33$ years. The *first* and *third* columns assume faster waning of severity-blocking immunity, with the *first* column having $\frac{1}{\delta_{V}}+\frac{1}{\delta_{V,\text{sev}}}=\frac{1}{\delta }+\frac{1}{\delta_{\text{sev}}}=0.5$ years and the *third* column having $\frac{1}{\delta_{V}}+\frac{1}{\delta_{V,\text{sev}}}=1.5$ years (since the vaccine is more durable) and $\frac{1}{\delta }+\frac{1}{\delta_{\text{sev}}}=0.5$ years. On the other hand, the *second* and *fourth* columns assume slower waning of severity-blocking immunity, with the *second* column having $\frac{1}{\delta }+\frac{1}{\delta_{\text{sev}}}=1.5$ years and $\frac{1}{\delta_{V}}+\frac{1}{\delta_{V,\text{sev}}}=2$ years, and the *fourth* column having $\frac{1}{\delta }+\frac{1}{\delta_{\text{sev}}}=1.5$ years and $\frac{1}{\delta_{V}}+\frac{1}{\delta_{V,\text{sev}}}=3$ years. In each panel, the left and right axes of the top plot are *I*_*w*_ and the fraction $\frac{I_{w}}{I_{P}+I_{S}+I_{w}}$, respectively, and the area plot colours correspond to the compartments in Fig 1B. In all panels, *ε* = 0.8. All other parameters are as in Figs 2 and 3, and the colours in the area plots are as in Fig 1B.

So far, we have examined changes in severity dynamics via total and relative infection levels across a range of settings for the strength of immunity and durations of both severity-blocking and transmission-blocking immunity. In Fig 4, we summarize synoptic medium-term immuno-epidemiological scenarios based on optimistic or pessimistic assumptions on severity-blocking immunity, different vaccination rates, and changes in durability of vaccines. For each scenario, we present time series of infections after severity-blocking immunity has waned and of the fraction of infections that these consist of. Below, we illustrate immunity and infection phenotypes over time. Note that at the bottom of each such area plot are the three infection types (*I*_*P*_, *I*_*S*_, and *I*_*w*_), and thus the total fraction of individuals infected is immediately seen visually. While we had previously assumed in Fig 2 (for existing vaccines) that the durations of vaccinal and natural severity-blocking immunity were equal, we now relax this assumption in optimistic scenarios for waning of severity-blocking immunity (*i.e*. second column of Fig 4) and assume in those settings that the average duration of vaccinal severity-blocking immunity is (optimistically) slightly longer than that of natural severity-blocking immunity (2 years instead of 1.5 years). For a more durable vaccine, we assume that transmission-blocking immunity lasts on average 1.33 years, and that severity-blocking immunity lasts on average either 1.5 years (in the more pessimistic scenario with faster waning) or 3 years (if waning is optimistically slower).

With a low vaccination rate, vaccinal characteristics have limited impact on immuno-epidemiological dynamics (compare left two plots and right two plots, *top row*, Fig 4). However, the relative time scale of waning severity-blocking immunity drastically alters the immune landscape (*top row*, Fig 4). With an intermediate vaccination rate, a durable vaccine has important dynamical impacts (as also seen in [20, 22]) (*middle row*, Fig 4). Intermediate vaccination rates also partially modulate pessimistic outcomes if severity wanes rapidly; this is further emphasized if vaccination is increased further (Fig 4). However, if severity-blocking immunity wanes rapidly and a vaccine does not provide long-lasting transmission-blocking protection, then the buildup of susceptibles with waned severity-blocking immunity remains substantial irrespective of vaccination rates (compare leftmost plots of each row, Fig 4). Thus, to decrease this accumulation, a high vaccination rate with a more durable vaccine is necessary.

### Heterogeneities in vaccination coverage

Current vaccination rates are very variable globally and at local scales, both due to inequity in supply and hesitancy. For example, uptake of bivalent booster doses in the United States has been low, even among those who received the initial vaccines [31]. In a specific region, inequity in supply can arise from a number of issues, including due to vaccine nationalism by other regions [21], and vaccine hesitancy can emerge from underlying behavioural drivers [32, 33]. As shown and discussed in previous work, these heterogeneities in vaccination can have important immuno-epidemiological impacts on the medium- and long-term dynamics of SARS-CoV-2 (e.g. [6, 34]).

**Fig 5:**
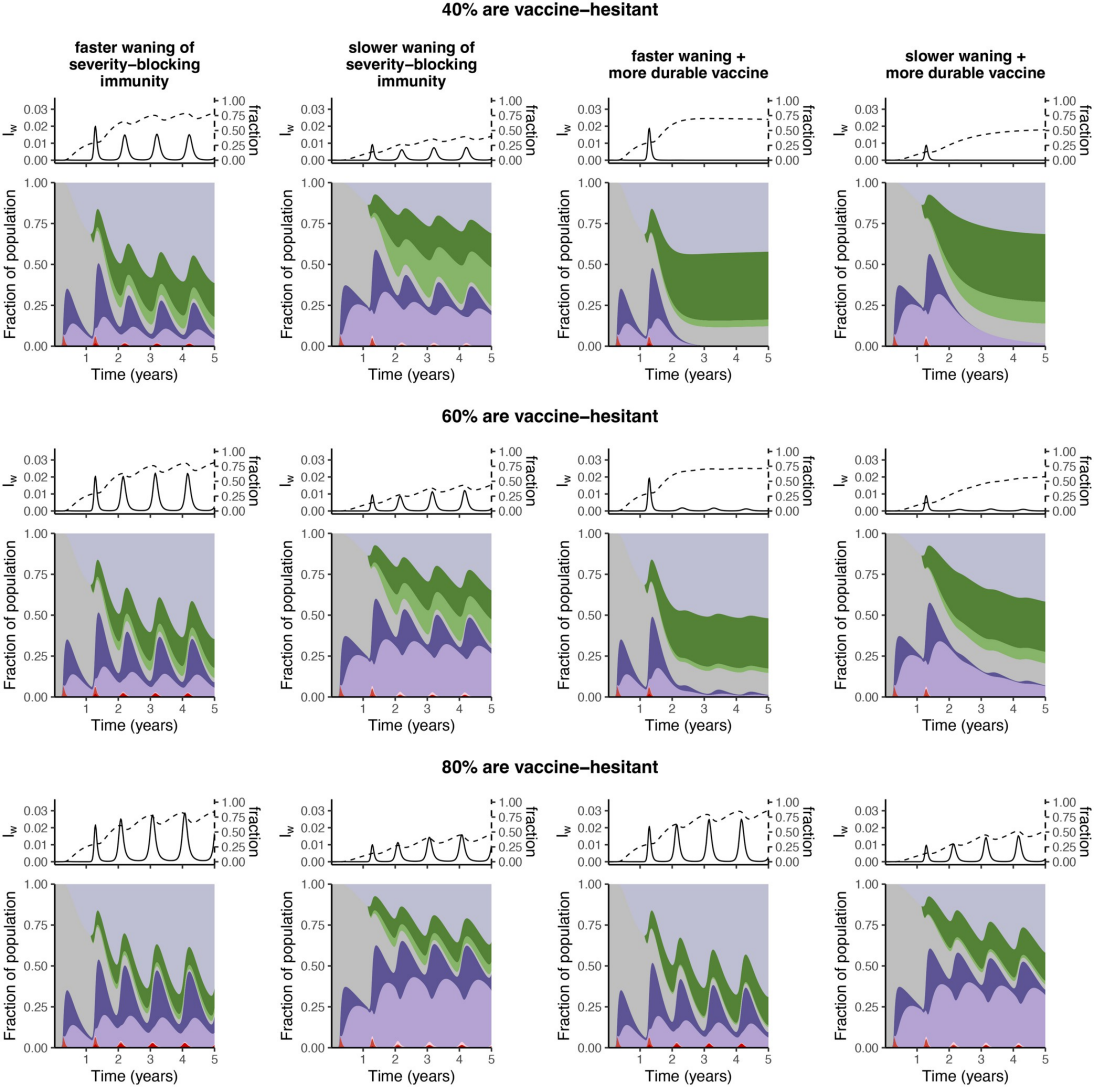
Synoptic landscapes with vaccine heterogeneities, caused by either unequal access or hesitancy. We assume a 2% weekly vaccination rate (*c.f*.*bottom row*, Fig 4), and keep the average vaccination rate constant across each row so that the vaccination rate among vaccine-adopters is *ν*, where *νN*_1_ = 0.02 (*N*_1_ = 1 − *N*_2_ is the fraction of vaccine adopters, and *N*_2_ is the fraction of individuals that are vaccine-hesitant). The columnar scenarios are as in those of Fig 4.

To investigate the potential consequences of vaccination heterogeneity on medium-term immune landscapes and burden due to infections after waning of severity-blocking immunity, we consider a simple extension of our basic framework with the addition of a group whose individuals never receive vaccinations, but otherwise mix homogeneously with individuals that are in the vaccine-adopter group (see S1 Text, *electronic supplementary materials*, for model equations). In Fig 5, we illustrate the medium-term outcomes for a range of vaccine-hesitant group sizes (*rows*), for different scenarios of severity-blocking immunity and vaccine characteristics (*columns*). In all these panels, we assume that the average vaccination rate is 2% per week (*i.e*. *νN*_1_ = 0.02, where *N*_1_ = 1 − *N*_2_ is the fraction of individuals that are vaccine-adopters and *N*_2_ is the fraction of individuals that are never vaccinated), which corresponds to a ‘high’ vaccination scenario in the homogeneous setting of Fig 4.

Direct comparisons between Fig 4 (*bottom row*) and the rows of Fig 5 reveal that the homogeneous vaccination assumption is an optimistic upper bound. In particular, vaccination heterogeneity increases the fraction of infections after severity-blocking immunity has waned, and can even lead to recurrent outbreaks if there are very few individuals that are receiving vaccinations. Intuitively, these observations emerge because vaccine-adopters are a heavily vaccinated group of individuals who are re-vaccinated often. On the other hand, individuals with waned immunity that are never vaccinated can only regain immunity via infection. Thus, even in an optimistic scenario where there is slower waning of severity-blocking immunity, a large fraction of these never-vaccinated individuals have waned severity-blocking immunity. Furthermore, if there is a substantial fraction of individuals that are not receiving vaccination, a more durable vaccine has almost no immuno-epidemiological effect on the population-level dynamics (compare left two columns with right two columns, respectively, of Fig 5, *bottom row*). Finally, if the average vaccination rate decreases, the impact of the resulting vaccine heterogeneity on immuno-epidemiological outcomes is slightly attenuated (*e.g*., see S1 Fig, *electronic supplementary materials* where the average rate is 0.01 per week).

**Fig 6:**
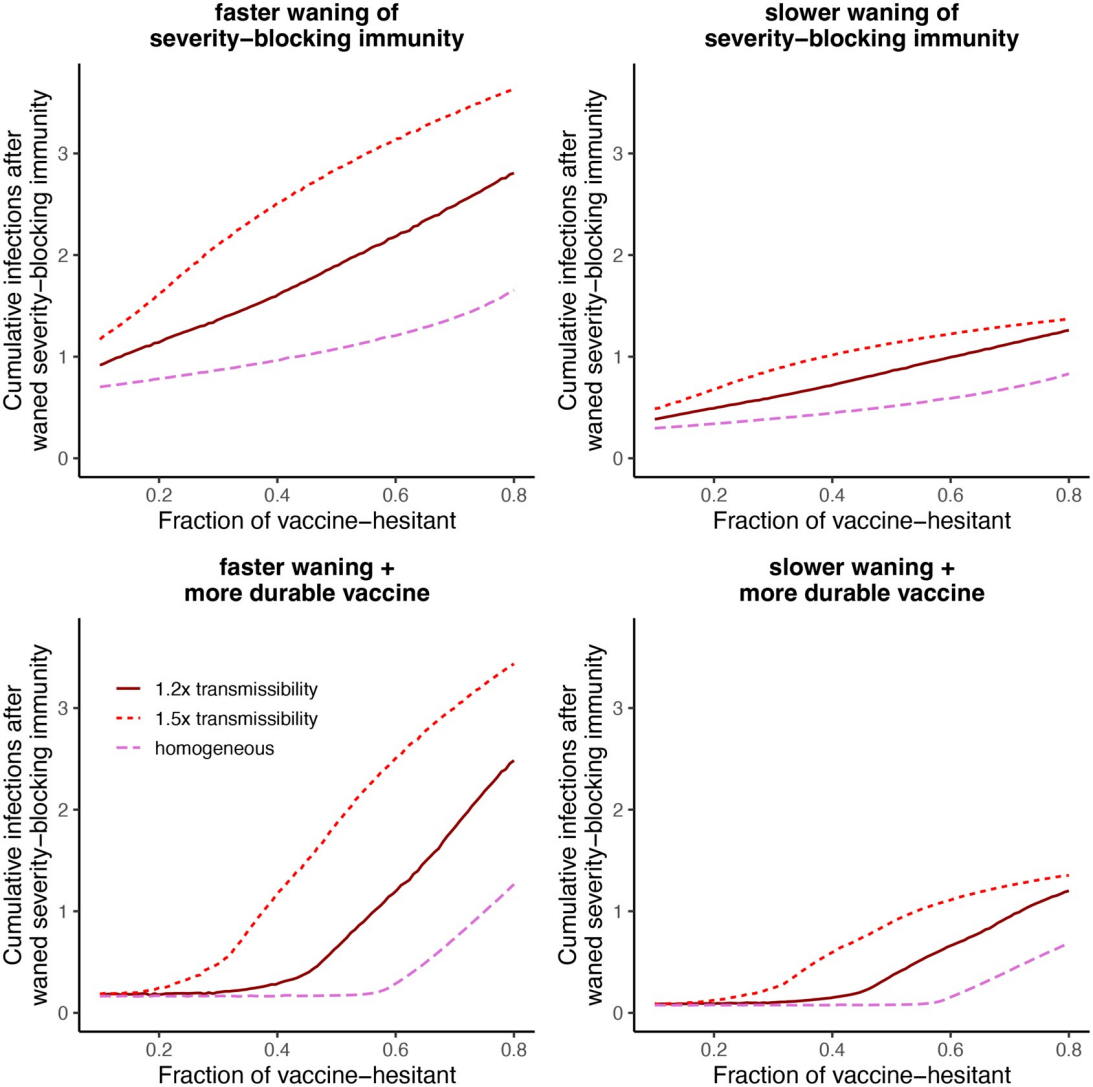
Cumulative infections with waned severity-blocking immunity after the onset of vaccination up to year 5 as a function of the fraction of individuals that are vaccine-hesitant. The *top left*, *top right*, *bottom left*, and *bottom right* panel depict the same scenarios as the first, second, third, and fourth columns of Figs 4 and 5, respectively. As in Fig 5, the average vaccination rate is constant. In each panel, the different lines denote different relative transmissibility values for vaccine hesitants.

In Fig 6, we examine the cumulative number of infections in *I*_*w*_ that occur after the onset of vaccination up to year 5 (relative to population size), as a function of the fraction of individuals that are vaccine-hesitant. As in Fig 5, we assume that the average vaccination rate is 2% per week. Across scenarios, an increase in vaccine hesitancy leads to a greater number of infections in *I*_*w*_. This effect is further magnified if severity-blocking immunity wanes rapidly (compare *left* with *right* panels of Fig 6, or if vaccine-hesitants have a higher transmissibility. As illustrated in Fig 5, vaccine hesitancy counters the deployment of a more durable vaccine (*bottom* panels, Fig 6). In particular, sufficient vaccine hesitancy can lead to a sharp increase in the cumulative number of infections in *I*_*w*_ (*bottom left* panel, Fig 6). Thus, if the fraction of individuals that are vaccine-hesitant is below this threshold, there are very few infections with waned severity-blocking immunity. However, if the fraction of individuals that are vaccine-hesitant increases further beyond this threshold, the cumulative number is substantially increased. If vaccine-hesitants have a higher transmissibility, we find that this threshold occurs for a much smaller level of vaccine hesitancy (*bottom left panel*, Fig 6), which can impact immuno-epidemiological dynamics (see S2 Fig, *electronic supplementary materials* for an example). Overall, these results illustrate that, even with corresponding adjustments to vaccination rates among adopters, vaccine hesitancy can substantially hinder the epidemiological benefits of more durable vaccines.

## Caveats and future directions

To distill the impacts of severity-blocking immunity on potential medium-term outcomes, we have made a number of simplifying assumptions in our modelling framework that should be relaxed in future work. First, we have ignored vaccine dosing regimes (see *e.g*. [20]) and assumed that individuals get vaccinated at some rate, with each subsequent vaccine they obtain giving rise to similar vaccinal immunity. In reality, multiple doses can lead to more robust immunity, and incorporating explicit vaccine doses in our model could reveal subsequent impacts. Relatedly, we have ignored the potential accumulation of immunity (whether transmission-blocking or against severity) after multiple exposures. Combining this refined model with that of [22] could elucidate any intricacies that may emerge due to the interaction between accumulating immunity and waning severity-blocking immunity. We have also ignored the impact of time-dependent variable vaccination rates, and incorporating this with specific vaccination data for various regions would be valuable. Notwithstanding these complexities, we have shown that the qualitative impact of hesitancy on vaccine performance is robust to underlying assumptions. This underlines the importance of more refined and granular models for dynamics of hesitancy in future work.

While we have examined heterogeneities in vaccination, we have ignored various other heterogeneities, *e.g*. in transmission [35, 36], or due to age [15] or vulnerabilities [37]. Exploring these further, and their confluence with vaccination heterogeneities and severity-blocking immunity, is an important future direction. In particular, we have assumed that interactions between individuals that are vaccine-hesitant and those that adopt vaccines are homogeneous. In reality, however, interactions within a group could be more likely than between groups. These features could enhance transmission potential and reduce the likelihood of control via vaccination (see *e.g*. [6] for a simple consideration of this), and examining the interplay between these effects and waning severity-blocking immunity is an important are of future research.

Relatedly, we have ignored the dynamics of human behavior [38], especially regarding adherence to nonpharmaceutical interventions (*e.g*. [39, 40]) or vaccination [41, 42]. However, the potential feedbacks between these social and epidemiological dynamics could shape immuno-epidemiological trajectories. Thus, incorporating these features into an epidemiological-behavioral model with severity-blocking immunity would be particularly fruitful.

We have also omitted individual variations in viral loads and immune kinetics. In particular, it would be particularly insightful to formulate cross-scale models that couple our framework with within-host dynamics. Coupled with a model for viral evolution, such a framework could potentially aid in understanding viral phylodynamics of SARS-CoV-2 (see [29, 43, 44]). Relatedly, we have ignored the dynamics of Long COVID, and exploring the connections between this, severity-blocking immunity, and potential medium-term chronic burden is a salient avenue for future work. Overall, understanding the impacts of these various heterogeneities will require complex models with comprehensive data (*e.g*. from large cohort studies [29]).

In line with previous work (see *e.g*. [6, 20–22, 45]) we have assumed that NPIs decrease transmission by a fixed value for fixed periods of time. However, NPIs are often implemented dynamically. Furthermore, in the absence of mandated NPIs, individuals may still choose to adhere to certain interventions (*e.g*. mask-wearing, social distancing). Incorporating the underlying social dynamics that then determine NPI adherence in such a setting may be important [39]. Thus, a potentially fruitful future avenue would be to couple our simple immuno-epidemiological models with more realistic formulations of NPI adherence, calibrated to particular regions of interest.

The impacts of vaccination, transmission-blocking and severity-blocking immunity, vaccine hesitancy, varying periods of NPIs, and climatic effects on transmission can be further explored using the interactive online application at https://grenfelllab.shinyapps.io/covid19immunity/.

## Conclusion

As the SARS-CoV-2 outbreak continues to progress, testing and monitoring of infections has been widely relaxed and the public health emergency of international concern (PHEIC) has ended, but transmission remains high. In parallel, while the mass of data accumulated so far has improved our understanding of host immune responses following infection or vaccination, a number of uncertainties remain, especially in the duration of immunity against severe disease and in the relative susceptibility to reinfection after waning of transmission-blocking immunity.

Our simple models reveal that a large range of outcomes can emerge from uncertainties in both the duration of severity-blocking immunity and the strength of immunity, and from the confluence of these two parameters. In particular, our findings emphasize that the strength of immunity shapes immuno-epidemiological dynamics at multiple resolutions, and that the duration of severity-blocking immunity has a major effect on population-level immune landscapes and potential burdens. Thus, to properly infer infection dynamics from hospitalization data, accurate estimates of both these parameters are needed, which could be accomplished via future cohort studies monitoring immuno-epidemiology [29] and a Global Immunological Observatory [26–28].

Finally, we have also shown that high vaccination rates, in combination with a more durable vaccine, can alleviate pessimistic outcomes for both the buildup of susceptible individuals with waned severity-blocking immunity and for the level of infections with waned severity-blocking immunity. Our results also illustrate the importance of broad vaccination coverage, echoing previous findings that argued for equities in vaccination access [20–22, 34]. In particular, we find that ignoring the specter of vaccine hesitancy in regions awash with vaccines can, at the population-level, essentially counteract important pharmaceutical developments to improve vaccine breadth and strength. Since we have shown that this result is generally robust to model assumptions on underlying uncertainties of severity-blocking immunity, our work underlines the need to identify and understand the behavioural drivers of vaccine hesitancy [33]. In tandem, since the impact of hesitancy is especially amplified if vaccine-hesitants have higher transmissibility, our results further stress the importance of nonpharmaceutical interventions in regions with elevated levels of hesitancy. Overall, to prevent pessimistic outcomes from waning severity-blocking immunity, increases in global vaccination rates in conjunction with the development of more robust vaccines are necessary.

## Supporting information

S1 Fig Vaccine hesitancy with a lower baseline vaccination rate. This figure is as in Fig 5, but with an average vaccination rate of 1% per week (instead of the 2% per week in Fig 5).
(PDF)

S2 Fig The impacts of increased transmissibility of vaccine hesitants on immuno-epidemiological dynamics. This figure is as in Fig 5, but with *α*_*V*_ = 1.2.
(PDF)

S1 Text Supplementary information text. (PDF)

S1 File Zip file with R code. (ZIP)
