# Supplementary material for: Impact of waning immunity against SARS-CoV-2 severity exacerbated by vaccine hesitancy
Source: PLoS Comput Biol. 2024 Aug 5;20(8):e1012211. doi: 10.1371/journal.pcbi.1012211 (PMC11299835; doi:10.1371/journal.pcbi.1012211)
Supplement: S1 Text — (PDF) [file pcbi.1012211.s003.pdf]

Supporting Information Text for: *Impact of waning immunity against SARS-CoV-2 severity exacerbated by vaccine hesitancy*

Chadi M. Saad-Roy[1,2,*] Sinead E. Morris[3] Mike Boots[2,4]
Rachel E. Baker[5] Bryan L. Lewis[6] Jeremy Farrar[7]
Madhav V. Marathe[6,8] Andrea L. Graham[9] Simon A. Levin[9]
Caroline E. Wagner[10] C. Jessica E. Metcalf[9,11] Bryan T. Grenfell[9,11]

[1]Miller Institute for Basic Research in Science, University of California, Berkeley
[2]Department of Integrative Biology, University of California, Berkeley
[3] Department of Pathology and Cell Biology, Columbia University Medical Center, Columbia University
[4] Department of Biosciences, University of Exeter
[5] Department of Epidemiology, Brown School of Public Health, Brown University
[6] Network Systems Science and Advanced Computing Division, Biocomplexity Institute, University of Virginia
[7] The Wellcome Trust, UK
[8] Department of Computer Science, University of Virginia
[9] Department of Ecology and Evolutionary Biology, Princeton University
[10] Department of Bioengineering, McGill University
[11]School of Public and International Affairs, Princeton University
[*]csaadroy@berkeley.edu

Model with vaccine hesitancy

To incorporate heterogeneity in vaccination, we consider two groups, one in which individuals are receiving vaccinations, and the other in which individuals are never vaccinated. We assume that the mixing between both groups is homogeneous, and denote the group by the numbered subscript on the state variables. Furthermore, α_V denotes the relative transmissibility of vaccine hesitants.

Therefore, the model becomes:

$$\frac{dS_{P,1}}{dt} = \mu N_1 - \beta(t)S_{P,1}(I_{P,1} + \alpha I_{S,1} + \alpha I_{w,1} + \alpha_V(I_{P,2} + \alpha I_{S,2} + \alpha I_{w,2})) - \mu S_{P,1} - s_{\text{vax}}\nu S_{P,1}, \tag{1a}$$

$$\frac{dI_{P,1}}{dt} = \beta(t)S_{P,1}(I_{P,1} + \alpha I_{S,1} + \alpha I_{w,1} + \alpha_V(I_{P,2} + \alpha I_{S,2} + \alpha I_{w,2})) - (\gamma + \mu)I_{P,1}, \tag{1b}$$

$$\frac{dR_1}{dt} = \gamma(I_{P,1} + I_{S,1} + I_{w,1}) - (\delta + \mu)R_1, \tag{1c}$$

$$\frac{dS_{S,1}}{dt} = \delta R_1 - \varepsilon\beta(t)S_{S,1}(I_{P,1} + \alpha I_{S,1} + \alpha I_{w,1} + \alpha_V(I_{P,2} + \alpha I_{S,2} + \alpha I_{w,2})) - \mu S_{S,1} - s_{\text{vax}}\nu S_{S,1} - \delta_{\text{sev}} S_{S,1}, \tag{1d}$$

$$\frac{dI_{S,1}}{dt} = \varepsilon\beta(t)S_{S,1}(I_{P,1} + \alpha I_{S,1} + \alpha I_{w,1} + \alpha_V(I_{P,2} + \alpha I_{S,2} + \alpha I_{w,2})) + \varepsilon\beta(t)S_{V,1}(I_{P,1} + \alpha I_{S,1} + \alpha I_{w,1} + \alpha_V(I_{P,2} + \alpha I_{S,2} + \alpha I_{w,2})) - (\gamma + \mu)I_{S,1}, \tag{1e}$$

$$\frac{dV_1}{dt} = s_{\text{vax}}\nu(S_{P,1} + S_{S,1} + S_{w,1} + S_{V,1}) - (\mu + \delta_V)V_1, \tag{1f}$$

$$\frac{dS_{V,1}}{dt} = \delta_V V_1 - \varepsilon\beta(t)S_{V,1}(I_{P,1} + \alpha I_{S,1} + \alpha I_{w,1} + \alpha_V(I_{P,2} + \alpha I_{S,2} + \alpha I_{w,2})) - (\delta_{V,\text{sev}} + \mu + s_{\text{vax}}\nu)S_{V,1}, \tag{1g}$$

$$\frac{dS_{w,1}}{dt} = \delta_{V,\text{sev}} S_{V,1} + \delta_{\text{sev}} S_{S,1} - \varepsilon\beta(t)S_{w,1}(I_{P,1} + \alpha I_{S,1} + \alpha I_{w,1} + \alpha_V(I_{P,2} + \alpha I_{S,2} + \alpha I_{w,2})) - \mu S_{w,1} - s_{\text{vax}}\nu S_{w,1}, \tag{1h}$$

$$\frac{dI_{w,1}}{dt} = \varepsilon\beta(t)S_{w,1}(I_{P,1} + \alpha I_{S,1} + \alpha I_{w,1} + \alpha_V(I_{P,2} + \alpha I_{S,2} + \alpha I_{w,2})) - (\mu + \gamma)I_{w,1}. \tag{1i}$$

$$\frac{dS_{P,2}}{dt} = \mu N_2 - \beta(t)S_{P,2}(I_{P,1} + \alpha I_{S,1} + \alpha I_{w,1} + \alpha_V(I_{P,2} + \alpha I_{S,2} + \alpha I_{w,2})) - \mu S_{P,2}, \tag{1j}$$

$$\frac{dI_{P,2}}{dt} = \beta(t)S_{P,2}(I_{P,1} + \alpha I_{S,1} + \alpha I_{w,1} + \alpha_V(I_{P,2} + \alpha I_{S,2} + \alpha I_{w,2})) - (\gamma + \mu)I_{P,2}, \tag{1k}$$

$$\frac{dR_2}{dt} = \gamma(I_{P,2} + I_{S,2} + I_{w,2}) - (\delta + \mu)R_2, \tag{1l}$$

$$\frac{dS_{S,2}}{dt} = \delta R_2 - \varepsilon\beta(t)S_{S,2}(I_{P,1} + \alpha I_{S,1} + \alpha I_{w,1} + \alpha_V(I_{P,2} + \alpha I_{S,2} + \alpha I_{w,2})) - \mu S_{S,2} - \delta_{\text{sev}} S_{S,2}, \tag{1m}$$

$$\frac{dI_{S,2}}{dt} = \varepsilon\beta(t)S_{S,2}(I_{P,1} + \alpha I_{S,1} + \alpha I_{w,1} + \alpha_V(I_{P,2} + \alpha I_{S,2} + \alpha I_{w,2})) - (\gamma + \mu)I_{S,2}, \tag{1n}$$

$$\frac{dS_{w,2}}{dt} = \delta_{\text{sev}}S_{S,2} - \varepsilon\beta(t)S_{w,2}(I_{P,1} + \alpha I_{S,1} + \alpha I_{w,1} + \alpha_V(I_{P,2} + \alpha I_{S,2} + \alpha I_{w,2})) - \mu S_{w,2}, \tag{1o}$$

$$\frac{dI_{w,2}}{dt} = \varepsilon\beta(t)S_{w,2}(I_{P,1} + \alpha I_{S,1} + \alpha I_{w,1} + \alpha_V(I_{P,2} + \alpha I_{S,2} + \alpha I_{w,2})) - (\mu + \gamma)I_{w,2}. \tag{1p}$$
