# Supplementary material for: Impact of waning immunity against SARS-CoV-2 severity exacerbated by vaccine hesitancy
Source: PLoS Comput Biol. 2024 Aug 5;20(8):e1012211. doi: 10.1371/journal.pcbi.1012211 (PMC11299835; doi:10.1371/journal.pcbi.1012211)
Supplement: S2 Fig — This figure is as in Fig 5, but with αV = 1.2. (PDF) [file pcbi.1012211.s002.pdf]

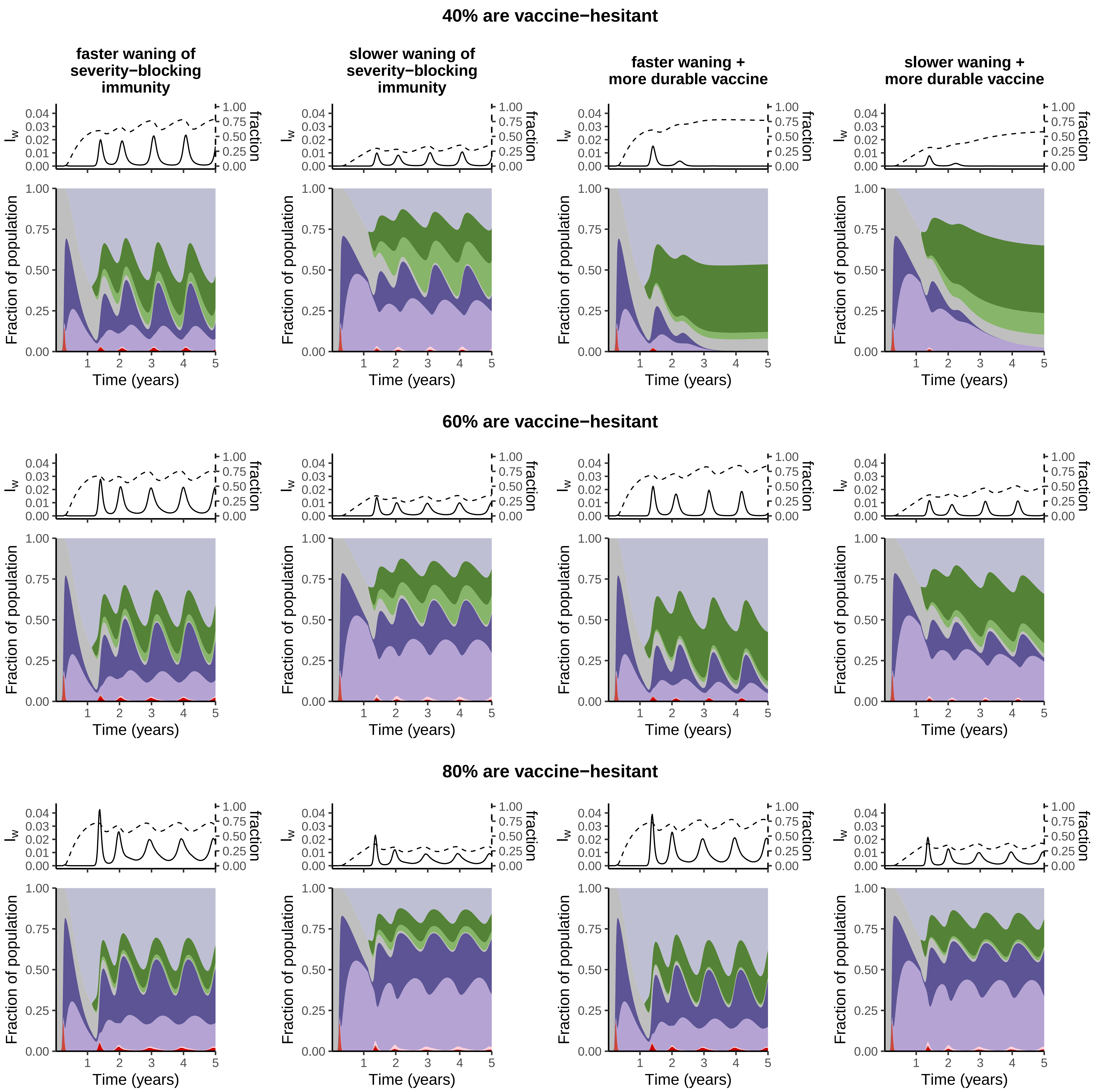

40% are vaccine−hesitant
60% are vaccine−hesitant
80% are vaccine−hesitant
faster waning of severity−blocking immunity
slower waning of severity−blocking immunity
faster waning + more durable vaccine
slower waning + more durable vaccine
I_W
fraction
Fraction of population
Time (years)
0.00
0.01
0.02
0.03
0.04
0.25
0.50
0.75
1.00
1
2
3
4
5
